# Differential effect on mortality of the timing of initiation of renal replacement therapy according to the criteria used to diagnose acute kidney injury: an IDEAL-ICU substudy

**DOI:** 10.1186/s13054-023-04602-7

**Published:** 2023-08-17

**Authors:** Saber Davide Barbar, Abderrahmane Bourredjem, Rémi Trusson, Auguste Dargent, Christine Binquet, Jean-Pierre Quenot, IDEAL-ICU Study, Raphaël Clere-Jehl, Romain Hernu, Florent Montini, Rémi Bruyère, Christine Lebert, Julien Bohé, Julio Badie, Jean-Pierre Eraldi, Jean-Philippe Rigaud, Bruno Levy, Shidasp Siami, Guillaume Louis, Lila Bouadma, Jean-Michel Constantin, Emmanuelle Mercier, Kada Klouche, Damien Du Cheyron, Gaël Piton, Djillali Annane, Samir Jaber, Therry van der Linden, Gilles Blasco, Jean-Paul Mira, Carole Schwebel, Loïc Chimot, Philippe Guiot, Mai-Anh Nay, Ferhat Meziani, Julie Helms, Claire Roger, Benjamin Louart

**Affiliations:** 1 Unité de Réanimation Médicale, Service des Réanimations, Centre Hospitalier Universitaire de Nîmes, Hôpital Caremeau, Place du Prof Robert Debré, 30029 Nîmes, France; 2 Université de Montpellier, Montpellier, France; 3 CIC 1432, Epidémiologie Clinique, Centre Hospitalier Universitaire Dijon-Bourgogne, BP 1541, Dijon, France; 4 Medical Intensive Care Unit, Hospices Civils de Lyon, Hôpital Lyon-Sud, 69437 Lyon, France; 5 Service de Médecine Intensive Réanimation, CHU Dijon, Dijon, France; 6 Lipness Team, INSERM Research Center LNC-UMR1231 and LabExLipSTIC, Université de Bourgogne, Dijon, France

**Keywords:** Renal replacement therapy, Kidney failure, Intensive care unit, Septic shock

## Abstract

**Background:**

This substudy of the randomized IDEAL-ICU trial assessed whether the timing of renal replacement therapy (RRT) initiation has a differential effect on 90-day mortality, according to the criteria used to diagnose acute kidney injury (AKI), in patients with early-stage septic shock.

**Methods:**

Three groups were considered according to the criterion defining AKI: creatinine elevation only (group 1), reduced urinary output only (group 2), creatinine elevation plus reduced urinary output (group 3). Primary outcome was 90-day all-cause death. Secondary endpoints were RRT-free days, RRT dependence and renal function at discharge. We assessed the interaction between RRT strategy (early vs. delayed) and group, and the association between RRT strategy and mortality in each group by logistic regression.

**Results:**

Of 488 patients enrolled, 205 (42%) patients were in group 1, 174 (35%) in group 2, and 100 (20%) in group 3. The effect of RRT initiation strategy on 90-day mortality across groups showed significant heterogeneity (adjusted interaction *p* = 0.021). Mortality was 58% vs. 42% for early vs. late RRT initiation, respectively, in group 1 (*p* = 0.028); 57% vs. 67%, respectively, in group 2 (*p* = 0.18); and 58% vs. 55%, respectively, in group 3 (*p* = 0.79). There was no significant difference in secondary outcomes.

**Conclusion:**

The timing of RRT initiation has a differential impact on outcome according to AKI diagnostic criteria. In patients with elevated creatinine only, early RRT initiation was associated with significantly increased mortality. In patients with reduced urine output only, late RRT initiation was associated with a nonsignificant, 10% absolute increase in mortality.

**Supplementary Information:**

The online version contains supplementary material available at 10.1186/s13054-023-04602-7.

## Key points

*Question:* Can acute kidney injury (AKI) diagnostic criteria modify the impact of the timing of renal replacement therapy (RRT)?

*Findings:* In this post hoc analysis of a randomized clinical trial that included 488 adults, the effect of RRT initiation strategy on 90-day mortality across groups showed significant heterogeneity. Mortality was 58% vs. 42% for early vs late RRT initiation in the creatinine elevation only group, a significant difference.

*Meaning:* The timing of RRT initiation has a differential impact on outcome according to AKI diagnostic criteria.

**Supplementary Information:**

The online version contains supplementary material available at 10.1186/s13054-023-04602-7.

## Introduction

Despite the frequent use of renal replacement therapy (RRT) in the intensive care units (ICUs) for critically ill patients with severe acute kidney injury (AKI), precise criteria for initiation of RRT are still not available. In the absence of life-threatening complications, clinicians have long been uncertain about when to initiate renal replacement therapy. Theoretically, earlier RRT initiation could improve electrolyte and acid–base control as well as fluid balance [1].

Three recent multicenter randomized controlled trials including a total of more than 4000 patients have explored the question of the timing of RRT initiation, namely the AKIKI (Artificial Kidney Initiation in Kidney Injury) trial [2], the IDEAL-ICU trial (Initiation of Dialysis Early Versus Delayed in the Intensive Care Unit) [3] and the STARRT-AKI trial (Timing of Initiation of Renal Replacement Therapy in Acute Kidney Injury) [4]. Although the inclusion criteria of these three studies differed slightly, they were all based on the KDIGO criteria [5].

The main message to emerge from the results of these three studies is that, in the absence of emergency criteria (such as hyperkalemia, metabolic acidosis or acute lung edema), there is no benefit in terms of mortality to be gained from immediate initiation of RRT in patients with KDIGO stage 2–3 AKI. On the contrary, deferring initiation of RRT enabled many patient to avoid RRT (between 38 [3, 4] and 49% [2]) and to spontaneously recover renal function in most cases. Thus, it may be argued that, instead of evaluating the effect of the timing of RRT initiation, these studies actually tested the potential for a KDIGO criteria-based strategy for RRT initiation. Taken together, the results of the three trials call into question the ability of the KDIGO criteria to predict the need for RRT and raise question about whether they should be used to decide on RRT initiation.

The question of the benefit to be gained from early RRT initiation remains open. The conclusions of these studies may have been shaped by the choice of inclusion criteria, which did not correctly identify the patients with the most severe forms of renal failure and, thus, those who might potentially benefit most from earlier initiation of RRT. We hypothesized that the severity of AKI is different in patients in whom AKI diagnosis was based solely on creatinine elevation, compared to patients in whom AKI is diagnosed on the basis of a reduction of urine output alone, or those with both criteria. We further hypothesize that the impact of early initiation of RRT may be different across these groups.

In this context, we performed a post hoc subanalysis among patient from the randomized IDEAL-ICU trial, to assess whether there is a differential effect of the timing of RRT initiation on 90-day mortality, according to the KDIGO criteria used to diagnose acute kidney injury (AKI), in patients with early-stage septic shock.

## Methods

### Study design and patients

This *post hoc* substudy used the data from the IDEAL-ICU trial (NCT016882590), a multicenter, randomized clinical trial conducted in 29 ICUs in France. Patients with severe AKI (stage F of the RIFLE classification [6]) and septic shock [7] were randomly assigned (1:1) to either an early or a delayed RRT initiation strategy [4]. The detailed protocol and results are available elsewhere [3, 8].

The failure (F) stage of the RIFLE classification, which was used in the IDEAL-ICU study, corresponds to stage 3 of the KDIGO classification, and is the preferred term used hereafter [9].

For the present analysis, we defined three groups, according to the criteria that had been used to define AKI and qualify patients for inclusion in IDEAL-ICU: patients who were enrolled solely on the basis of creatinine elevation (group 1), patients who were enrolled solely on the basis of reduced urinary output (oliguria or anuria – group 2) and patients who presented both criteria (group 3).

The original trial was approved by the competent French legal authorities, and the ethics committee *“Comité de Protection des Personnes Est 1”* (under the number 2012-A00519-34) for all participating centers. Written informed consent was obtained from the patient or a surrogate either before randomization or as soon as possible thereafter.

### Study interventions

In the early-strategy group, RRT was started within 12 h after the diagnosis of AKI. In the delayed strategy group, RRT was started 48 h after the diagnosis of AKI, or as soon as possible if at least one of the following pre-specified emergency criteria occurred before 48 h: hyperkalemia (serum potassium level > 6.5 mmol/liter), metabolic acidosis (pH < 7.15) or fluid overload refractory to diuretics with pulmonary edema.

The choice of RRT technique (intermittent or continuous) was at the discretion of each study site, and investigators were encouraged to follow international guidelines.

### Endpoints

The primary outcome was death from any cause at 90 days after randomization.

Secondary endpoints included ICU and hospital mortality, ICU and hospital length of stay, RRT-free days, RRT dependence at hospital discharge, and renal function in surviving patients who were not RRT dependent at hospital discharge (creatinine absolute values and creatinine progression, expressed as percentage change in creatinine values from baseline values).

In the present analysis, we evaluated the criteria for emergency renal replacement therapy within 7 days after randomization in all 3 groups, notably: severe metabolic acidosis (defined as a pH less than 7.15 and a base deficit of more than 5 mmol per liter or a bicarbonate level of 18 mmol or less per liter), severe hyperkalemia (defined as a potassium level of more than 6.5 mmol per liter with characteristic electrocardiographic changes) and fluid overload (defined as extravascular fluid overload that was refractory to diuretics, with pulmonary edema).

### Statistical analysis

Analysis comparing the early vs delayed RRT randomized arms (RRT timing) was performed according to the intention-to-treat principle. Giving the negligible number of patients lost of follow-up (11/488 = 2%) [3], death at 90 days was considered as a binary outcome. Death percentages were calculated according to the timing of RRT initiation, in each group (group 1: creatinine elevation only; group 2: reduced urine output only; group 3: both).

A logistic regression model, with the 90-day mortality as an independent variable, was used to evaluate the interaction term between RRT strategy and groups and to assess the association between death and RRT strategy in each group. Results are expressed as odds ratios (ORs) with 95% confidence intervals (CIs). In the presence of a significant interaction, a multivariate model, stratified by center and adjusted for major prognostic factors (age, SOFA score, immunosuppression, presence of cirrhosis, type of infection, presence of chronic kidney injury), was then performed to confirm the significance of the interaction and to estimate the effect of the timing of RRT in each group.

Patient characteristics are described according to group and RRT timing. Categorical variables are expressed as number and percentage and were compared using the Chi square or Fisher’s exact test, as appropriate. Continuous variables are expressed as means ± standard deviation (SD) or medians and interquartiles (Quartile (Q) 1 and Q3), and were compared using analysis of variance (ANOVA) or the Kruskall-Wallis test, as appropriate.

Secondary outcomes are described across groups according to the timing of RRT initiation. The proportion of patients with severe metabolic disorders or/and pulmonary edema due to fluid overload in the 7 days after randomization, ICU mortality, hospital mortality and RRT dependence were estimated. The ICU length of stay, number of ICU days free of RRT, variation in creatinine relative to the basal level were recorded for surviving, non-RRT-dependent patients at hospital discharge, as well as the hospital length of stay, and are expressed as medians and interquartile range.

Analyses were performed with SAS software, version 9.4 (SAS Institute Inc., Cary, NC). All p-values are two-sided, and the significance level was set at 0.05 for all analyses.

## Results

### Patient characteristics

Of the 488 patients enrolled in the trial, data were missing for 9 patients, and thus, 479 were included in the final analysis: 205 (42%) patients were included in group 1 (creatinine elevation only), 174 (35.5%) in group 2 (reduced urinary output only) and 100 (20.5%) in group 3 (creatinine elevation plus reduced urinary output).

The comparison of the baseline characteristics across groups is shown in Table 1. The comparisons of baseline characteristics between patients randomized to the early and those randomized to the delayed RRT initiation strategies in each group are given in the Additional file 1 (group 1 in Table S1, group 2 in Table S2 and group 3 in Table S3).

The comparison of the baseline characteristics across groups is shown in Table 1. The comparisons of baseline characteristics between patients randomized to the early and those randomized to the delayed RRT initiation strategies in each group are given in the Additional file 1 (group 1 in Table S1, group 2 in Table S2 and group 3 in Table S3).

**Table 1:** Patient characteristics according to the criterion used to diagnose AKI (IDEAL-ICU trial)

Characteristic	Group 1 creatinine elevation only (*N* = 205)	Group 2 reduced urinary output only (*N* = 100)	Group 3 creatinine elevation plus reduced urinary output (*N* = 174)	*P*-Value
Age (years)	69.5 ± 11.5	70.0 ± 12.2	66.6 ± 13.3	0.070
Sex, *n* (%)				0.110
Male	125 (61%)	113 (65%)	52 (52%)	
Female	80 (39%)	61 (35%)	48 (48%)	
BMI (Kg/m^2^)	28.8 ± 7.8	29.0 ± 7.7	29.2 ± 9.0	0.900
Coexisting conditions, *n* (%)				
Chronic renal failure	20 (10%)	43 (25%)	11 (11%)	< .001
Hypertension	126 (61%)	97 (56%)	56 (56%)	0.470
Diabetes	69 (34%)	51 (29%)	25 (25%)	0.290
Congestive heart failure	10 (5%)	20 (11%)	9 (9%)	0.060
Chronic respiratory failure	10 (5%)	11 (6%)	7 (7%)	0.710
Chronic liver disease	13 (6%)	31 (18%)	16 (16%)	0.002
Immunosuppression	55 (27%)	53 (30%)	33 (33%)	0.500
Septic shock infection type, *n* (%)				0.035
Community-acquired	150 (73%)	106 (61%)	70 (70%)	
Nosocomial	55 (27%)	68 (39%)	30 (30%)	
SAPS II at ICU admission	63.1 ± 15.3	66.3 ± 17.0	65.3 ± 14.8	0.140
SOFA score at randomization	11.8 ± 2.7	12.5 ± 3.0	12.8 ± 3.1	0.005
Exposure to at least one nephrotoxic agent within 4 days before randomization, *n* (%)	97 (47%)	83 (48%)	51 (51%)	0.820
Multiple organ support in ICU, *n* (%)				
Invasive mechanical ventilation	176 (86%)	162 (93%)	86 (86%)	0.060
Vasopressor support with norepinephrine or epinephrine	205 (100%)	174 (100%)	100 (100%)	
Inotropic support with dobutamine	48 (23%)	35 (20%)	23 (23%)	0.720
Extracorporeal membrane oxygenation	1 (0%)	5 (3%)	4 (4%)	0.090
Diagnostic criteria for acute kidney injury at the failure stage of the RIFLE classification, *n* (%)				
Oliguria	0	102 (59%)	64 (64%)	
Anuria	0	104 (60%)	67 (67%)	
Serum creatinine 3 times the baseline level	205 (100%)	0	100 (100%)	
Serum creatinine before ICU admission (µmol/l)	84.9 ± 40.2	103.9 ± 47.9	83.5 ± 39.7	< .001
Serum creatinine at enrollment (µmol/l)	335.1 ± 139.4	216.9 ± 84.6	337.6 ± 146.8	< .001
Blood urea nitrogen (mmol/l)	24.4 ± 9.9	17.7 ± 8.5	23.5 ± 11.2	< .001
Serum potassium (mEq/l ou mmol/l)	4.4 ± 0.9	4.4 ± 0.8	4.5 ± 0.9	0.820
Serum bicarbonate (mmol/l)	16.9 ± 4.5	18.0 ± 4.2	17.9 ± 4.5	0.038
Fluid balance before enrollment (ml/24 h)	2797.4 ± 2210.4	3335.0 ± 2286.1	3692.1 ± 2654.1	0.013

There were significant differences between groups in the baseline characteristics (Table 1): patients in group 3 more frequently had chronic renal failure and chronic liver disease, more frequently had nosocomial infection, and more frequently had higher creatinine before ICU admission but lower creatinine at enrollment. SOFA score at admission was significantly different among the three groups, lowest in group 1 and in group 2. Fluid balance was significantly different across the 3 groups, lowest in group 1 and highest in group 2. We did not observe any relevant differences between the early and delayed arms within each of the 3 groups (Additional file 1: Tables S1, S2 and S3).

### Primary endpoint

We found significant heterogeneity in the effect of RRT initiation strategy on 90 day mortality across groups (Table 2) (test for interaction: *p* = 0.048). In group 1, 57/99 patients (58%) died in the early RRT initiation group vs. 42/100 (42%) in the delayed strategy group (*p* = 0.028). In group 2, mortality was 48/84 (57%) vs. 59/88 (67%), respectively (*p* = 0.18), and in group 3, 29/50 (58%) vs. 26/47 (55%), respectively (*p* = 0.79), yielding odds ratios (ORs) of 1.87 (95% CI 1.07–3.29) in group 1, 0.65 (95%-CI 0.35, 1.22) in group 2, and 1.15 (95% CI 0.50, 2.49) in group 3, for early vs delayed RRT.

**Table 2:** Univariate analysis of 90-day mortality (primary endpoint – IDEAL-ICU trial)

	Randomization Arm				Effect of timing arm on 90-day mortality		Unadjusted *p*-value for interaction
	Early RRT (*N* = 246)		Delayed RRT (*N* = 242)		OR [95% CI]	*p*-value	
	*N*	Death at D90^#^	*N*	Death at D90^#^			
AKI qualifying criterion*							0.048
Group 1: Creatinine elevation only	104	57/99 (58%)	101	42/100 (42%)	1.87 [1.07, 3.29]	0.028	
Group 2: Reduced urinary output only	84	48/84 (57%)	90	59/88 (67%)	0.65 [0.35, 1.22]	0.18	
Group 3: both criteria	52	29/50 (58%)	48	26/47 (55%)	1.15 [0.50, 2.49]	0.79	

^*^9 patients with missing values on the qualifying criterion for AKI (6 in the early arm and 3 in the delayed arm)

^#^ 7 patients lost to follow-up in the early arm and 4 in the delayed arm

**Fig. 1:**
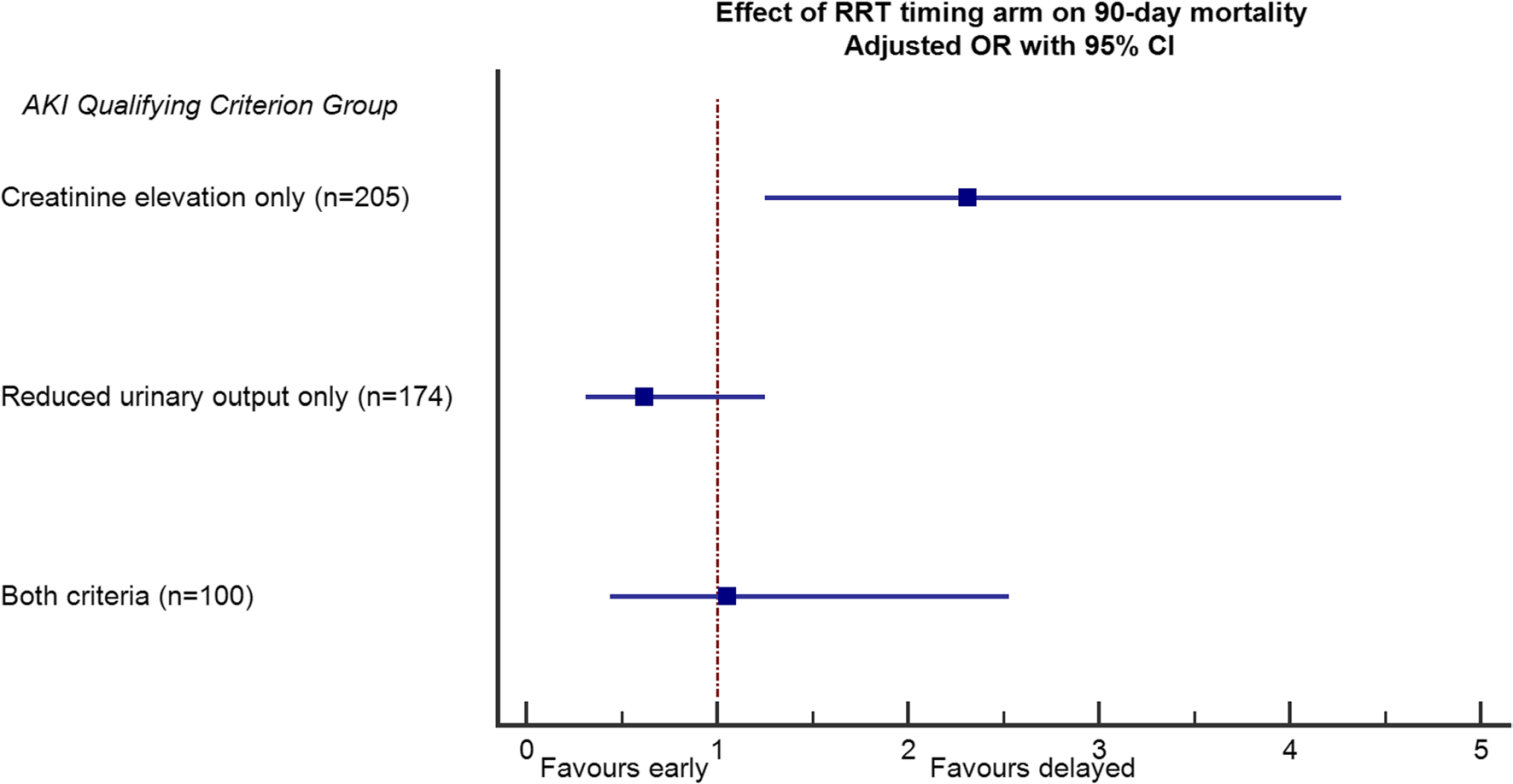
Multivariate analysis of the association between RRT timing arm with 90-day mortality according to the qualifying criterion for AKI, in the IDEAL-ICU trial. (Interaction *p*-value = 0.021; adjustment covariates: cirrhosis, age, immunodepression, SOFA at randomization, nosocomial infection and chronic kidney injury)

By multivariate analysis, stratified by center, and adjusted for age, pre-existing immunosuppression or cirrhosis, hospital acquired infection, SOFA score and chronic kidney injury, the results of the interaction test remained unchanged, signaling significant heterogeneity (p-value from adjusted test of interaction, *p* = 0.021). Early RRT was associated with a significantly increased risk of 90-day death in group 1 (creatinine elevation only) (OR 2.31, 95% CI 1.25, 4.27, *p* = 0.006) (Table 3, Fig. 1).

**Table 3:** Multivariate analysis of the factors associated with 90-day mortality (primary endpoint – IDEAL-ICU trial)

Effect	Interaction	OR	95% CI	*p*-value
RRT early vs. delayed				0.021*
	Creatinine elevation only	2.31	[1.25, 4.27]	0.006
	Reduced urinary output only	0.62	[0.31, 1.25]	0.18
	Creatinine elevation + reduced urinary output	1.05	[0.44, 2.53]	0.36
Cirrhosis		2.96	[1.43, 6.10]	0.003
Age (per 10 additional yrs)		1.48	[1.22, 1.79]	< .0001
Immunodepression		1.76	[1.10, 2.83]	0.02
SOFA at randomization				0.01
11–13 vs. 6–10		1.37	[0.84, 2.26]	0.21
14–21 vs. 6–10		2.39	[1.35, 4.24]	0.003
Nosocomial infection		1.74	[1.10, 2.76]	0.02
Chronic kidney injury		1.29	[0.71, 2.33]	0.40

*Adjusted *p*-value for interaction: (RRT timing)*(Groups by AKI qualifying criterion)

### Secondary endpoints

The results of the secondary endpoints across the three groups are given in Table 4, according to early or delayed RRT initiation. In group 1, ICU mortality was numerically higher in the early RRT arm, while mortality was numerically higher in the delayed RRT arm in group 2, with no difference in group 3.

**Table 4:** Secondary endpoints (IDEAL-ICU trial)

	Group 1		Group 2		Group 3	
	Early RRT (*N* = 104)	Delayed RRT (*N* = 101)	Early RRT (*N* = 84)	Delayed RRT (*N* = 90)	Early RRT (*N* = 52)	Delayed RRT (*N* = 48)
Median ICU days (IQR)	9 (4–15)	10 (5–16)	12 (6–23)	10 (5–20)	11 (5–27.5)	11.5 (4.5–26)
Median RRT-free days in ICU (IQR)	4.5 (2–9)	6 (4–11)	6 (3–12)	6.5 (4–13)	4 (2–8.5)	3.5 (2–8)
ICU mortality, *n* (%)	42 (40%)	33 (33%)	42 (50%)	50 (56%)	24 (46%)	22 (46%)
Median hospital days (IQR)	19.5 (9–34.5)	24 (11–45)	25 (10–44.5)	20.5 (10–38)	27.5 (11.5–41.5)	25.5 (13.5–46)
State at hospital discharge, *n* (%)						
Unknown	6 (6%)	3 (3%)	4 (5%)	3 (3%)	0	4 (8%)
Death	55 (53%)	40 (40%)	46 (55%)	56 (62%)	26 (50%)	26 (54%)
RRT dependence	1 (1%)	1 (1%)	0	2 (2%)	3 (6%)	1 (2%)
RRT-free	42 (40%)	57 (56%)	34 (40%)	29 (32%)	23 (44%)	17 (35%)
Metabolic acidosis, *n* (%)	13 (13%)	20 (20%)	4 (5%)	12 (13%)	5 (10%)	8 (17%)
Hyperkalemia, *n* (%)	0	5 (5%)	0	2 (2%)	0	2 (4%)
Fluid overload, *n* (%)	1 (1%)	4 (4%)	0	3 (3%)	0	2 (4%)
Creatinine variation for RRT-free patients at hospital discharge*						
Creat basal level (µmol/l)	76.9 ± 15.28	83.56 ± 44.84	103.44 ± 62.81	105.11 ± 32.47	79.63 ± 28.75	79.06 ± 25.77
Creat at hospital discharge (µmol/l)	108.58 ± 64.15	103.83 ± 55.58	118.93 ± 76.62	145.81 ± 102.63	114.27 ± 71.72	152.8 ± 139.2

There was no difference in any of the three groups between early and delayed strategies in terms of ICU length of stay or RRT-free days. The criteria for emergency renal replacement therapy in the 7 days after randomization (severe metabolic acidosis, severe hyperkalemia and fluid overload) were numerically more frequent in the delayed arm in all 3 groups.

## Discussion

To the best of our knowledge, this is the first study based on a randomized clinical trial to find a differential impact on outcome of the timing of RRT initiation, according to the diagnostic criteria used to define AKI. Our results indicate that patients in the failure category of the RIFLE classification (corresponding to KDIGO stage 3) are a heterogeneous group, whereby those diagnosed on the basis of creatinine elevation alone do not appear to benefit from early initiation of RRT, but on the contrary, may actually be at risk for increased mortality with such a strategy. Conversely, patients diagnosed on the basis of reduced urine output only had a numerically, albeit non-statistically significantly higher mortality rate with delayed RRT initiation.

At the time the IDEAL-ICU study was designed, in the absence of consensual criteria for RRT initiation, the definition of “early” versus “delayed” or “late” initiation of RRT was arbitrary. Thus, we chose the RIFLE criteria for the early-strategy group of IDEAL-ICU [3] (a similar choice was made using the KDIGO criteria in the 2 other recent RCTs [2, 4]) because the RIFLE criteria are simple, easy to assess in clinical practice, pragmatic and correlated with outcomes [10]. However, these criteria were not developed with the intention of being used to trigger RRT initiation and have not been validated for this purpose. Conversely, a retrospective observational study that reviewed critically ill patients with severe AKI (RIFLE stage F) showed that about one third of them did not receive RRT and suggested that using the RIFLE score as a trigger for RRT initiation was unlikely to improve outcomes [11].

The decision to initiate RRT is usually made in a wider clinical context that takes account of the patient’s comorbidities and other organ dysfunctions. Clinicians do not decide solely on the basis of RIFLE/KDIGO criteria, and it is important to stress that our results concern a population with septic shock and acute renal failure, with invasive mechanical ventilation in over 85% of cases, and therefore with failure of three organs in most patients. Nevertheless, the RIFLE/KDIGO criteria remain major determinants of the decision to initiate RRT in critically ill patients in routine practice [12].

In fact, the RIFLE and KDIGO scores are both based on an increase in serum creatinine and a reduction in urine output [13], parameters that have well-known limitations. For serum creatinine, determination of the baseline level, the delayed peak value and the relationship with muscle mass are the principal limitations. For urine output, the lack of specificity [14] is the most important issue. Of note, in our study, hemodynamic optimization in the early phase of septic shock with repeated fluid challenge and the high volume of fluids received by patients before and up to 24 h after enrollment could have increased the specificity of this parameter for identifying severe AKI with a need for RRT. Our data do not provide an unequivocal formal explanation for the increased mortality observed with early initiation of RRT in patients included solely on the basis of creatinine elevation alone. However, several potential explanations may be put forward. A first hypothesis is that creatinine is a time-lagged marker of renal injury, and thus, after optimized resuscitation during the initial phase of AKI, renal function was already recovering spontaneously in these patients by the time of inclusion. The counterpart of this hypothesis is that patients diagnosed solely on the basis of reduced urine output, had a more constituted and severe form of AKI, which is therefore less rapidly reversible. In this scenario, earlier initiation of RRT could make sense. Indeed, in our analysis, we observed an absolute difference in mortality of 10% in this group, with higher mortality in those with late initiation of RRT. However, the difference was not statistically significant, precluding any definitive conclusions about the effect of RRT timing on mortality in these patients, even though an 10% absolute difference in mortality would likely be clinically meaningful. Moreover, patients in group 2 (urine output-based AKI) had more severe illness, based on higher SOFA score; they also had higher fluid balance, and it has previously been observed that earlier RRT initiation may benefit patients who have greater fluid overload [15]. The higher fluid balance in Group 2 (urine output-based AKI) compared to Group 1 (creatinine-based AKI) may be attributed to more aggressive fluid therapy in patients with reduced urinary output and could perhaps explain the severity of AKI in Group 2 and the potential (albeit non-statistically significant) benefit of earlier RRT initiation in this group.

This is not the first time that an increase in mortality has been observed with early RRT when initiation is based on creatinine level alone. In a prospective multicenter observational study [16] enrolling 1,238 patients, the timing of RRT was classified as “early” or “late” according to median creatinine at the time RRT was started. In that study, when stratified by creatinine values, late RRT initiation was associated with lower crude and covariate-adjusted mortality, in line with our results.

A further possible interpretation of our results is that the IDEAL-ICU trial [3], like other trials comparing early vs delayed RRT strategies in patients with severe AKI [2, 4], may have failed to identify differences in survival, as a result of mixing patients with divergent levels of risk. In a secondary analysis of the AKIKI and IDEAL-ICU trials [17], we provided proof-of-concept for the heterogeneity of treatment effects between the early and delayed strategies across levels of baseline risk, within 48 h after allocation to the delayed strategy. Specifically, in those allocated to delayed RRT initiation, patients with a low risk of RRT initiation within 48 h may benefit more from a delayed strategy, whereas those at intermediate-high risk of RRT initiation within 48 h likely benefit from an early initiation strategy.

An important strength of our study is that the data stem from a multicenter randomized controlled trial, in a large population of patients in the acute phase of septic shock. Second, the groups included in this analysis are fine-tuned, with specific criteria, providing a level of nuance not present in other studies on this topic. However, this study also has some limitations. Firstly, this is a *post hoc* analysis; however, our definition of the groups included in this analysis is coherent with the initial purpose of the study, and our results are not modified by multivariate analysis taking into account major confounding factors. Secondly, this analysis may suffer from a lack of statistical power, since the IDEAL-ICU was not designed for this purpose, and therefore, the results of the present analysis should be seen as hypothesis-generating. Our findings deserve further evaluation in specifically designed studies. Third, we have to consider the trigger for RRT initiation in the delayed arm. In our study, RRT was mandated 48 h after randomization for all patients in the delayed arm unless kidney recovery was evident. This delay may not be long enough for spontaneous recovery, especially in group 1, and, in any case, may not reflect actual clinical practice especially in light of recent publications [4]. Fourth, readers should be aware that in the IDEAL-ICU study, we did not implement any scales for predicting either worsening or persistent AKI, or early recovery of function, such as the furosemide stress test, and we also did not evaluate any other biomarker of renal function [18].

## Conclusions

In conclusion, the ideal timing for RRT initiation in the setting of severe AKI, despite the publication of high quality randomized controlled trials on this topic, remains a complex and partially unanswered question. Early initiation of RRT is associated with higher mortality in patients whose AKI was diagnosed solely on the basis of elevated serum creatinine. Our study shows that among critically ill patients with septic shock, mortality following early or delayed initiation of RRT is different, depending on the criteria used to diagnose AKI. This highlights a need for further research into the clinical and biological markers of AKI severity, and the indications for RRT. Future studies should aim to identify predictive factors of the need of RRT, in order to better select populations included in future trials about the timing of RRT.

### Supplementary Information

**Additional file 1.** Full List of Investigators of IDEAL-ICU Study Group. **Table S1:** Patient characteristics following the randomization arm in the “creatinine elevation only” sub-group. **Table S2:** Patients characteristics following randomization arm in the “creatinine elevation + reduced urinary output” sub-group. **Table S3:** Patient characteristics following randomization arm in the “reduced urinary output only” sub-group.

## Data Availability

All data generated or analyzed during this study are included in this published article [and its supplementary information files].
