# Supplementary material for: Differential effect on mortality of the timing of initiation of renal replacement therapy according to the criteria used to diagnose acute kidney injury: an IDEAL-ICU substudy
Source: Crit Care. 2023 Aug 17;27:316. doi: 10.1186/s13054-023-04602-7 (PMC10436583; doi:10.1186/s13054-023-04602-7)
Supplement: Supplementary file 1 — Additional file 1. Full List of Investigators of IDEAL-ICU Study Group. Table S1: Patient characteristics following the randomization arm in the “creatinine elevation only” sub-group. Table S2: Patients characteristics following randomization arm in the “creatinine elevation + reduced urinary output” sub-group. Table S3: Patient characteristics following randomization arm in the “reduced urinary output only” sub-group. [file 13054_2023_4602_MOESM1_ESM.doc]

**Supplementary Material For:**

**Differential effect on mortality of the timing of initiation of renal replacement therapy according to the criteria used to diagnose acute kidney injury: An IDEAL-ICU substudy.**

**Table of Contents:**

1. **Full List of Investigators**
2. **Table S1: Patient characteristics following the randomization arm in the “creatinine elevation only” sub-group**
3. **Table S2: Patients characteristics following randomization arm in the “creatinine elevation + reduced UO” sub-group**
4. **Table S3: Patient characteristics following randomization arm in the “reduced UO only” sub-group**

**Full List of Investigators of the IDEAL-ICU Study:**

Raphaël Clere-Jehl, M.D., Romain Hernu, M.D., Florent Montini, M.D., Rémi Bruyère, M.D., Christine Lebert, M.D., Julien Bohé, M.D., Ph.D., Julio Badie, M.D., Jean-Pierre Eraldi, M.D., Jean-Philippe Rigaud, M.D., Ph.D., Bruno Levy, M.D., PhD., Shidasp Siami, M.D., Ph.D., Guillaume Louis, M.D., Lila Bouadma, M.D., Ph.D., Jean-Michel Constantin, M.D., Ph.D., Emmanuelle Mercier, M.D., Kada Klouche, M.D., Ph.D., Damien Du Cheyron, M.D., Ph.D., Gaël Piton, M.D., Ph.D., Djillali Annane, M.D., Ph.D., Samir Jaber, M.D., PhD., Therry van der Linden, M.D., Ph.D., Gilles Blasco, M.D., Jean-Paul Mira, M.D., Ph.D., Carole Schwebel, M.D., Ph.D., , Loïc Chimot M.D., Philippe Guiot, M.D., Mai-Anh Nay, M.D., Ferhat Meziani, M.D., Ph.D., Julie Helms, M.D., Ph.D., Claire Roger, M.D., Ph.D., Benjamin Louart, M.D.

**Affiliations:**

B Louart: Réanimation Médicale, Centre Hospitalier Universitaire de Nîmes, Université de Montpellier, France.

RCJ, FM and JH: Université de Strasbourg, Faculté de médecine, Hôpitaux Universitaires de Strasbourg, Service de réanimation, Nouvel Hôpital Civil, Strasbourg, France.

RH: Réanimation Médicale, Hôpital Edouard Herriot, Hospices Civils de Lyon, Lyon, France.

FMo: Réanimation Polyvalente, Centre Hospitalier d’Avignon, Avignon, France.

RB: Réanimation Polyvalente, Centre Hospitalier de Bourg-en Bresse, Bourg-en Bresse, France.

CL: Service de Médecine Intensive-Réanimation, Centre Hospitalier de La Roche sur Yon, La Roche sur Yon, France.

J Bohe: Intensive Care Unit, Hospices Civils de Lyon, Centre Hospitalier Lyon-Sud, Pierre Bénite, France.

J Badie: Réanimation Polyvalente, Hôpital Nord Franche-Comté, Belfort, France.

JPE and JPR: Médecine Intensive Réanimation, Centre Hospitalier de Dieppe, France.

B Levy: Service de Réanimation Médicale, Centre Hospitalier Universitaire Nancy Brabois, Nancy, France.

SS: Service d'anesthésie-réanimation, Centre Hospitalier d'Etampes, 26 avenue Charles De Gaulle, Etampes, France.

GL: Réanimation Polyvalente, Hôpital Bon Secours, Centre Hospitalier Régional de Metz, Metz, France.

LB: UMR 1137-IAME Team 5-DeScID (Decision Science in Infectious Diseases), Control and care and INSERM Université Paris Diderot, Sorbonne Paris Cité, F-75018 Paris, and Medical and Infectious Diseases ICU; Bichat–Claude-Bernard Hospital, Assistance Publique–Hôpitaux de Paris, Paris, France.

JMC: Pole de Médecine PériOpératoire, GReD; UMR/CNRS6293; UCA; INSERM U1103, CHU Clermont-Ferrand, 1 place Lucie et Raymond Aubrac, 63003 Clermont-Ferrand, France.

EM: Service de Réanimation Médicale, Centre Hospitalier Universitaire Régional de Tours, Tours, France.

KK: Service de Réanimation Médicale, Centre Hospitalier Universitaire Lapeyronie, Montpellier, France.

DDC: CHU de Caen, Service de Réanimation Médicale, Caen, F-14000, France.

GP: Service de réanimation médicale, Centre Hospitalier Universitaire de Besançon, France.

DA: Service de médecine intensive et réanimation, hôpital Raymond Poincaré, 104 boulevard Raymond Poincaré, 92380 Garches, France and Laboratory of Infection & Inflammation U1173, University of Versailles SQY, INSERM, 2 avenue de la source de la Bièvre, 78180 Montigny-le-Bretonneux, France.

SJ: Saint Eloi Centre Hospitalier Universitaire de Montpellier, University of Montpellier and INSERM U1046, 34000 Montpellier, France.

TvdL: Service de Médecine Intensive Réanimation, Groupe des hôpitaux de l'Institut Catholique de Lille (GHICL), Université catholique de Lille, Lille, France.

GB: Service de Réanimation Chirurgicale, Centre Hospitalier Universitaire de Besançon, Besançon, France.

JPM: Service de Réanimation Médicale, Hôpital Cochin, Paris, France.

CS: Service de Réanimation Médicale, Centre Hospitalier Universitaire de Grenoble, Grenoble, France.

LC: Service de Réanimation, Centre Hospitalier de Perigueux, Perigueux, France.

PG: Service de Réanimation Polyvalente, Centre Hospitalier Général de Mulhouse, Mulhouse, France.

MAN: Médecine Intensive Réanimation, Centre Hospitalier Régional d’Orléans, 14 Avenue de l'Hôpital, CS 86809, 45067 Orléans Cedex 2, France.

CR: Réanimation Chirurgicale – Centre Hospitalier Universitaire de Nîmes, France.

| **Supplementary Material**  **Table S1: Patient characteristics following the randomization arm in the “creatinine elevation only” sub-group**   |  | | | | | | | --- | --- | --- | --- | --- | --- | | **Characteristic** | **Early Strategy (N=104)** | | **Delayed Strategy (N=101)** | | ***P* Value** | | Age (years) | 69.7 | +11.6 | 69.3 | +11.5 | 0.84 | | Sex, n (%) |  |  |  |  | 0.87 | | Male | 64 | (62%) | 61 | (60%) |  | | Female | 40 | (38%) | 40 | (40%) |  | | BMI (Kg/m2) | 28.5 | +7.0 | 29.1 | +8.7 | 0.59 | | Coexisting conditions, n (%) |  |  |  |  |  | | Chronic renal failure | 9 | (9%) | 11 | (11%) | 0.59 | | Hypertension | 66 | (63%) | 60 | (59%) | 0.55 | | Diabetes | 37 | (36%) | 32 | (32%) | 0.56 | | Congestive heart failure | 5 | (5%) | 5 | (5%) | 0.96 | | Chronic respiratory failure | 8 | (8%) | 2 | (2%) | 0.06 | | Chronic liver disease | 6 | (6%) | 7 | (7%) | 0.73 | | Immunosuppression | 25 | (24%) | 30 | (30%) | 0.36 | | Septic choc infection type, n (%) |  |  |  |  | 0.98 | | Community-acquired | 76 | (73%) | 74 | (73%) |  | | Nosocomial | 28 | (27%) | 27 | (27%) |  | | SAPS II at ICU admission | 62.8 | +15.1 | 63.4 | +15.6 | 0.80 | | SOFA score at randomization | 11.7 | +2.6 | 11.9 | +2.7 | 0.58 | | Exposure to at least one nephrotoxic agent within 4 days before randomization, n (%) | 51 | (49%) | 46 | (46%) | 0.62 | | Multiple organ support in ICU, n (%) |  |  |  |  |  | | Invasive mechanical ventilation | 88 | (85%) | 88 | (87%) | 0.61 | | Vasopressor support with norepinephrine or epinephrine | 104 | (100%) | 101 | (100%) |  | | Inotropic support with dobutamine | 24 | (23%) | 24 | (24%) | 0.91 | | Extracorporeal membrane oxygenation | 0 |  | 1 | (1%) | 0.31 | | Diagnostic criteria for acute kidney injury at the failure stage of the RIFLE classification, n (%) |  |  |  |  |  | | Oliguria | 0 |  | 0 |  |  | | Anuria | 0 |  | 0 |  |  | | Serum creatinine 3 times the baseline level | 104 | (100%) | 101 | (100%) |  | | Serum creatinine before ICU admission (µmol/l) | 83.5 | +42.9 | 86.3 | +37.4 | 0.62 | | Serum creatinine at enrollment (µmol/l) | 323.6 | +126.9 | 346.9 | +150.8 | 0.23 | | Blood urea nitrogen (mmol/l) | 23.7 | +9.2 | 25.1 | +10.5 | 0.30 | | Serum potassium (mEq/l ou mmol/l) | 4.3 | +0.8 | 4.5 | +0.9 | 0.28 | | Serum bicarbonate (mmol/l) | 17.0 | +4.5 | 16.8 | +4.4 | 0.84 | | Fluid balance before enrollment (ml/24 hr) | 2974.1 | +2405.8 | 2656.4 | +2044.2 | 0.37 | |
| --- | --- | --- | --- | --- | --- | --- | --- | --- | --- | --- | --- | --- | --- | --- | --- | --- | --- | --- | --- | --- | --- | --- | --- | --- | --- | --- | --- | --- | --- | --- | --- | --- | --- | --- | --- | --- | --- | --- | --- | --- | --- | --- | --- | --- | --- | --- | --- | --- | --- | --- | --- | --- | --- | --- | --- | --- | --- | --- | --- | --- | --- | --- | --- | --- | --- | --- | --- | --- | --- | --- | --- | --- | --- | --- | --- | --- | --- | --- | --- | --- | --- | --- | --- | --- | --- | --- | --- | --- | --- | --- | --- | --- | --- | --- | --- | --- | --- | --- | --- | --- | --- | --- | --- | --- | --- | --- | --- | --- | --- | --- | --- | --- | --- | --- | --- | --- | --- | --- | --- | --- | --- | --- | --- | --- | --- | --- | --- | --- | --- | --- | --- | --- | --- | --- | --- | --- | --- | --- | --- | --- | --- | --- | --- | --- | --- | --- | --- | --- | --- | --- | --- | --- | --- | --- | --- | --- | --- | --- | --- | --- | --- | --- | --- | --- | --- | --- | --- | --- | --- | --- | --- | --- | --- | --- | --- | --- | --- | --- | --- | --- | --- | --- | --- | --- | --- | --- | --- | --- | --- | --- | --- | --- | --- | --- | --- | --- | --- | --- | --- | --- | --- | --- | --- | --- | --- | --- | --- | --- | --- | --- | --- | --- | --- | --- | --- | --- |

| **Table S2: Patient characteristics following randomization arm in the “creatinine elevation + reduced UO” sub-group** |
| --- |

| **Randomization Arm** | | | | | |
| --- | --- | --- | --- | --- | --- |
| **Variable** | **Early strategy (N=52)** | | **Delayed strategy (N=48)** | | ***P* Value** |
| Age (years) | 66.7 | (11.4) | 66.4 | (15.2) | 0.92 |
| Age category, n (%) |  |  |  |  | 0.21 |
| . <60 years | 12 | (23%) | 16 | (33%) |  |
| . [60-80[ | 33 | (63%) | 22 | (46%) |  |
| . >=80 years | 7 | (13%) | 10 | (21%) |  |
| Sex, n (%) |  |  |  |  | 0.99 |
| Male | 27 | (52%) | 25 | (52%) |  |
| Female | 25 | (48%) | 23 | (48%) |  |
| BMI | 27.9 | (8.6) | 30.7 | (9.3) | 0.13 |
| Documented chronic renal failure, n (%) | 4 | (8%) | 7 | (15%) | 0.27 |
| Hypertension, n (%) | 28 | (54%) | 28 | (58%) | 0.65 |
| Diabetes, n (%) | 14 | (27%) | 11 | (23%) | 0.64 |
| Chronic heart failure (NYHA III/IV), n (%) | 6 | (12%) | 3 | (6%) | 0.36 |
| Documented chronic respiratory failure, n (%) | 5 | (10%) | 2 | (4%) | 0.30 |
| Cirrhosis (known or suspected), n (%) | 8 | (15%) | 8 | (17%) | 0.86 |
| Immunosuppression, n (%) | 19 | (37%) | 14 | (29%) | 0.43 |
| KNAUS : Health status in the 6 previous months |  |  |  |  | 0.61 |
| . Class A | 18 | (35%) | 16 | (34%) |  |
| . Class B | 19 | (37%) | 16 | (34%) |  |
| . Class C | 13 | (25%) | 10 | (21%) |  |
| . Class D | 2 | (4%) | 5 | (11%) |  |
| Knaus (C/D vs A/B), n (%) | 15 | (29%) | 15 | (32%) | 0.74 |
| Septic choc infection type, n (%) |  |  |  |  | 0.86 |
| Community-acquired | 36 | (69%) | 34 | (71%) |  |
| Nosocomial | 16 | (31%) | 14 | (29%) |  |
| SAPS II (first 24h ICU) | 66.4 | (16.5) | 64.1 | (12.7) | 0.45 |
| SOFA score at randomization | 12.9 | (3.2) | 12.8 | (3.0) | 0.88 |
| Exposure to at least one nephrotoxic agent within 4 days before randomization | 28 | (54%) | 23 | (48%) | 0.55 |
| Invasive mechanical ventilation, n (%) | 46 | (88%) | 40 | (83%) | 0.46 |
| Vasopressprs, n (%) | 52 | (100%) | 48 | (100%) | . |
| Inotropic agents, n (%) | 10 | (19%) | 13 | (27%) | 0.35 |
| ECMO, n (%) | 0 | (0%) | 4 | (8%) | 0.034 |
| Oliguria < 0.3 ml/kg/h over 24h, n (%) | 34 | (65%) | 30 | (63%) | 0.76 |
| Anuria for at least 12h, n (%) | 35 | (67%) | 32 | (67%) | 0.95 |
| Serum creatinine 3 times the baseline level | 52 | (100%) | 48 | (100%) | . |
| Baseline creatinine (µmol/l) | 82.8 | (35.8) | 84.3 | (44.0) | 0.85 |
| Creatinine at diagnosis (µmol/l) | 331.8 | (142.7) | 343.9 | (152.3) | 0.68 |
| Blood urea nitrogen (mmol/l) | 22.4 | (10.3) | 24.8 | (12.1) | 0.29 |
| Serum potassium (mEq/l ou mmol/l) | 4.3 | (0.9) | 4.6 | (1.0) | 0.10 |
| Serum bicarbonate (mmol/l) | 18.0 | (4.9) | 17.7 | (4.2) | 0.76 |
| Fluid balance on day 0 | 3644.8 | (2836.7) | 3732.7 | (2519.6) | 0.88 |

**Table S3: Patients characteristics following randomization arm in the “reduced UO only” sub-group**

| **Randomization Arm** | | | | | |
| --- | --- | --- | --- | --- | --- |
| **Variable** | **EER Précoce (N=84)** | | **EER Différée (N=90)** | | ***P* Value** |
| Age (years) | 70.4 | (11.7) | 69.6 | (12.7) | 0.66 |
| Age category, n (%) |  |  |  |  | 0.87 |
| . <60 years | 15 | (18%) | 18 | (20%) |  |
| . [60-80[ | 49 | (58%) | 49 | (54%) |  |
| . >=80 years | 20 | (24%) | 23 | (26%) |  |
| Sex, n (%) |  |  |  |  | 0.016 |
| Male | 47 | (56%) | 66 | (73%) |  |
| Female | 37 | (44%) | 24 | (27%) |  |
| BMI | 29.7 | (8.2) | 28.3 | (7.2) | 0.22 |
| Documented chronic renal failure, n (%) | 18 | (21%) | 25 | (28%) | 0.33 |
| Hypertension, n (%) | 49 | (58%) | 48 | (53%) | 0.51 |
| Diabetes, n (%) | 26 | (31%) | 25 | (28%) | 0.65 |
| Chronic heart failure (NYHA III/IV), n (%) | 8 | (10%) | 12 | (13%) | 0.43 |
| Documented chronic respiratory failure, n (%) | 5 | (6%) | 6 | (7%) | 0.85 |
| Cirrhosis (known or suspected), n (%) | 16 | (19%) | 15 | (17%) | 0.68 |
| Immunosuppression, n (%) | 23 | (27%) | 30 | (33%) | 0.39 |
| KNAUS : Health status in the 6 previous months |  |  |  |  | 0.71 |
| . Class A | 21 | (25%) | 26 | (29%) |  |
| . Class B | 30 | (36%) | 30 | (34%) |  |
| . Class C | 29 | (35%) | 26 | (29%) |  |
| . Class D | 4 | (5%) | 7 | (8%) |  |
| Knaus (C/D vs A/B), n (%) | 33 | (39%) | 33 | (37%) | 0.77 |
| Septic choc infection type, n (%) |  |  |  |  | 0.23 |
| Community-acquired | 55 | (65%) | 51 | (57%) |  |
| Nosocomial | 29 | (35%) | 39 | (43%) |  |
| SAPS II (first 24h ICU) | 66.6 | (18.2) | 65.9 | (16.0) | 0.79 |
| SOFA score at randomization | 12.3 | (3.1) | 12.6 | (3.0) | 0.48 |
| Exposure to at least one nephrotoxic agent within 4 days before randomization | 47 | (56%) | 36 | (40%) | 0.035 |
| Invasive mechanical ventilation, n (%) | 79 | (94%) | 83 | (92%) | 0.63 |
| Vasopressprs, n (%) | 84 | (100%) | 90 | (100%) | . |
| Inotropic agents, n (%) | 14 | (17%) | 21 | (23%) | 0.27 |
| ECMO, n (%) | 1 | (1%) | 4 | (4%) | 0.20 |
| Oliguria < 0.3 ml/kg/h over 24h, n (%) | 52 | (62%) | 50 | (56%) | 0.40 |
| Anuria for at least 12h, n (%) | 48 | (57%) | 56 | (62%) | 0.49 |
| Serum creatinine 3 times the baseline level | 0 | (0%) | 0 | (0%) | . |
| Baseline creatinine (µmol/l) | 99.2 | (47.9) | 108.4 | (47.8) | 0.21 |
| Creatinine at diagnosis (µmol/l) | 207.7 | (89.0) | 225.7 | (79.8) | 0.17 |
| Blood urea nitrogen (mmol/l) | 17.0 | (8.1) | 18.4 | (8.8) | 0.25 |
| Serum potassium (mEq/l ou mmol/l) | 4.4 | (0.9) | 4.4 | (0.7) | 0.58 |
| Serum bicarbonate (mmol/l) | 17.7 | (4.3) | 18.3 | (4.1) | 0.34 |
| Fluid balance on day 0 | 3279.6 | (2515.4) | 3373.4 | (2126.8) | 0.81 |
